# Type I collagen gene suppresses tumor growth and invasion of malignant human glioma cells

**DOI:** 10.1186/1475-2867-7-12

**Published:** 2007-06-20

**Authors:** Kimi Honma, Teruo Miyata, Takahiro Ochiya

**Affiliations:** 1 Section for Studies on Metastasis, National Cancer Center Research Institute, Tokyo, Japan; 2 Koken Bioscience Institute, Koken Co., Ltd., Tokyo, Japan

## Abstract

**Background:**

Invasion is a hallmark of a malignant tumor, such as a glioma, and the progression is followed by the interaction of tumor cells with an extracellular matrix (ECM). This study examined the role of type I collagen in the invasion of the malignant human glioma cell line T98G by the introduction of the human collagen type I α1 (HCOL1A1) gene.

**Results:**

The cells overexpressing HCOL1A1 were in a cluster, whereas the control cells were scattered. Overexpression of HCOL1A1 significantly suppressed the motility and invasion of the tumor cells. The glioma cell growth was markedly inhibited *in vitro *and *in vivo *by the overexpression of HCOL1A1; in particular, tumorigenicity completely regressed in nude mice. Furthermore, the HCOL1A1 gene induced apoptosis in glioma cells.

**Conclusion:**

These results indicate that HCOL1A1 have a suppressive biological function in glioma progression and that the introduction of HCOL1A1 provides the basis of a novel therapeutic approach for the treatment of malignant human glioma.

## Background

The processes of tumor cell invasion into the stromal tissue are closely related to the interactions between tumor cells and extracellular matrix (ECM). Furthermore, the alteration of the expression and modification of ECM proteins in tumor cells is relevant to their invasiveness into surrounding normal tissue. For example, loss of fibronectin, which is an ECM glycoprotein from the cell surface, has been indicated to be closely associated with malignant transformation of cells [1]. It was then shown that the overexpression of fibronectin in human fibrosarcoma cells was able to suppress the motility and growth potential of tumor cells [1]. Collagens are the most important components of ECM and play an important role in cell adhesion, movement, differentiation, proliferation, and metastasis of tumor cells. Recently, fragments of type IV, type XV, and type XVIII collagen, which are components of various basement membranes, have been extensively studied for their potential in the reduction of angiogenesis and tumorigenesis [2,3]. Endostatin, a non-collagenous C-terminal domain (NC1) fragment of type XVIII collagen, was the first endogenous fragment characterized with anti-angiogenic properties [4]. The NC1 fragments of type XV collagen and type IV collagen were also identified as an endogenous angiogenesis inhibitor [5-11].

Type I collagen is a fibrillar collagen that consists of two polypeptide chains, the α1(I) chain and the α2(I) chain. Most of type I collagen molecules are distributed as heterotrimers [α1(I)]_2_α2(I), and a small number of molecules exist as homotrimers [α1(I)]_3_. Although type I collagen is the most abundant collagen in skin, bone, and other tissues and organs, no type I collagen molecule and/or fragments have been demonstrated to have suppressive efficacy on malignant phenotypes of a tumor. However, variations in the production of type I collagen by tumors have been reported in the relationship with tumor progression, and transformed cells generally synthesize less collagen than their counterparts [12-15]. The differential expression between hepatocellular carcinomas and normal liver was revealed, and type I collagen was down-regulated in hepatocellular carcinomas [15]. In a neuroblastoma, type I collagen biosynthesis is a helpful marker for studying specific patterns of transdifferentiation associated with the loss of malignant potential [16]. In addition, type I collagen was able to inhibit growth and malignant transformation in human glioma cells [17,18]. In the present study, a type I collagen gene was introduced to glioma, which is characterized by marked tumor cell proliferation and extensive local invasion, in order to examine the inhibitory effects of the overexpressed HCOL1A1 on invasion and tumorigenesis. The results showed that overexpression of HCOL1A1 in malignant glioma cells suppressed cell proliferation, inhibited cell motility and invasiveness, and arrested tumor formation *in vivo*.

## Results

### Overexpression of HCOL1A1 in T98G cells

**Figure 1:**
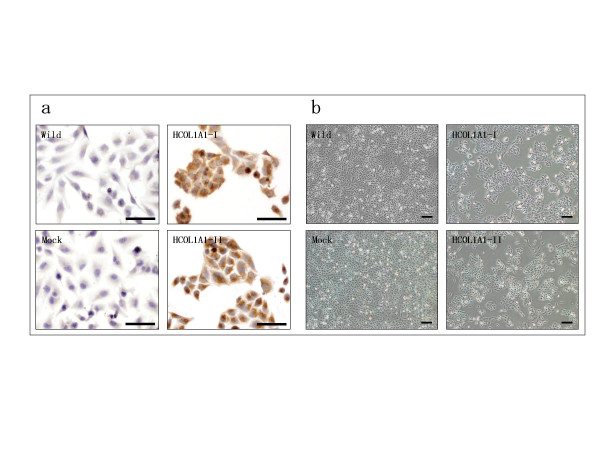
Morphological changes of the HCOL1A1-overexpressing T98G cells. Wild cells (Wild), control transfected cells (Mock), and HCOL1A1-transfected cells (HCOL1A1-I and HCOL1-II) were seeded at 1 × 10^5 ^cells on 6-cm culture dishes and grown for 2 to 4 days. (a) Immunocytochemical staining of T98G cells (day 2). HCOL1A1 peptides were immunostained with polyclonal anti-α1(I) collagen antibody. Scale bar: 100 μm. (b) Phase-contrast photomicrographs of T98G cells (day 4). Scale bar: 200 μm.

Human type I collagen cDNA (HCOL1A1) was transfected into the human malignant glioma cell line T98G. Twenty-nine colonies of G418-resistant cells were cloned, and G418-resistant clones were screened for expression of HCOL1A1 by immuno-detection with a polyclonal anti-α1(I) collagen antibody. Two independent clones stably over-expressing HCOL1A1, designated HOCL1A1-I and HCOL1A1-II, were isolated. By immunocytochemical staining HCOL1A1-I and HCOL1A1-II cells showed strong immunoreactivity, while HCOL1A1 peptides were not detectable in wild cells and Mock cells (Fig. 1a). Expression of HCOL1A1 peptides were also confirmed in the whole cell lysates and the conditioned medium from HCOL1A1-transfected cells by immunoblot analysis. Although immunoreactive protein bands, which are absent in parental wild cells and Mock-transfected cells, were clearly identified, HCOL1A1 peptides were digested into fragments and did not fold into triple helices characteristic of type I collagen (data not shown).

The cell densities of the HCOL1A1-transfected cells (4 days) were significantly different from that of wild cells and Mock cells (Fig. 1b): this was clearly shown that the growth inhibition of HCOL1A1-transfected cells, as well as they were tightly cell-cell contacted in clusters, while control cells were dispersed and spindle-shaped in scattering as usual (Fig. 1a). These results suggest that forced expression of HCOL1A1 resulted in apparent cell density changes in T98G cells.

### Inhibition of cell growth by HCOL1A1

**Figure 2:**
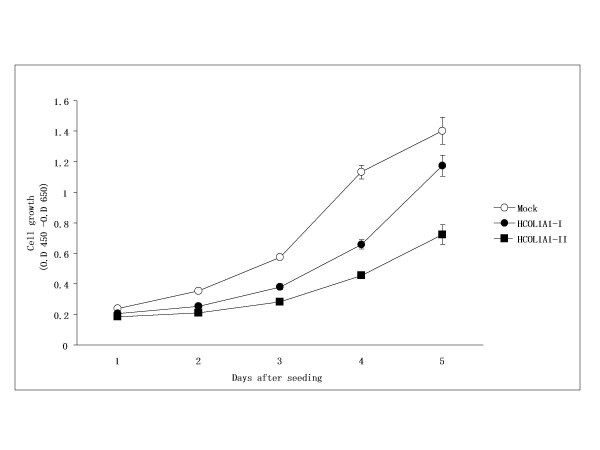
Inhibition of T98G cell growth *in vitro *by HCOL1A1 expression. The graph shows the growth curves of Mock cells (Mock, ○) and HCOL1A1-transfected cells (HCOL1A1-I, ● ; HCOL1A1-II, ■). The same number of cells were seeded and cultured for 5 days. At each time point, the cells were assayed for proliferation. Each value represents mean ± SD (n = 8).

The effect of HCOL1A1 expression on the growth of T98G cells was further evaluated kinetically (Fig. 2). Cell growth was significantly suppressed in HCOL1A1-I and HCOL1A1-II cells, *P *< 0.001 and *P *< 0.0001 versus Mock cells, respectively. These results showed that overexpression of HCOL1A1 was able to inhibit the growth of T98G cells *in vitro*.

### Suppression of tumor cell motility and invasion by HCOL1A1

**Figure 3:**
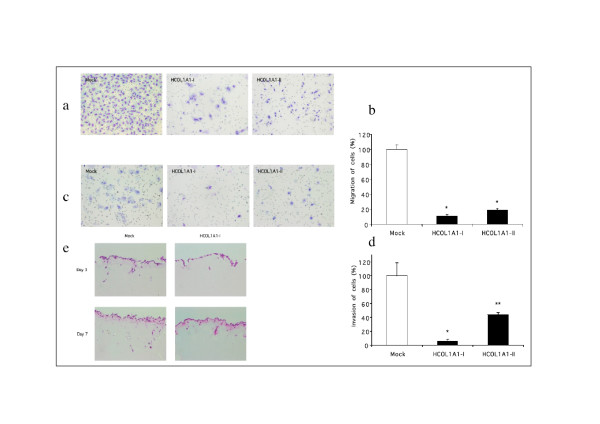
Suppression of T98G cell motility and invasion by overexpression of HCOL1A1. Mock cells (Mock) and HCOL1A1-transfected cells (HCOL1A1-I and HCOL1A1-II) were added to a transwell chamber and the cell motility was evaluated. (a) The migrated cells were stained and photographed under the microscope at × 100 magnification. (b) The number of migrated cells was counted, and the results represent a percentage of Mock cells. Each value is a mean ± SD (n = 4). *, *P *< 0.001, when tested against the Mock. Mock cells (Mock) and HCOL1A1-transfected cells (HCOL1A1-I and HCOL1A1-II) were added to a transwell chamber coated with Matrigel and the cell invasion was evaluated. (c) The invaded cells were stained and photographed under a microscope at × 100 magnification. (d) The number of invaded cells was counted, and the results are expressed as a percentage of Mock cells. Each value is a mean ± SD (n = 4). *, *P *< 0.001, **, *P *< 0.01 when tested against the Mock. The cell invasion into an *in vitro *invasion model was evaluated using reconstituted basement membrane wafers. (e) Mock cells (Mock) and HCOL1A1-transfected cells (HCOL1A1-I) were plated onto Matrigel wafers. On days 3 and 7 after plating, Matrigel wafers and adherent cells were fixed, and sections were stained with hematoxylin and eosin. Magnification, × 200.

Whether the HCOL1A1 peptide inhibits tumor cell motility was examined. (Fig. 3a, 3b). The motility of HCOL1A1-transfected cells was dramatically lower than that of Mock cells. The migration of HCOL1A1-I and HCOL1A1-II cells was inhibited more than 80% as compared to Mock cells.

Furthermore, the effect of HCOL1A1 overexpression on the invasiveness of T98G cells was assessed. Figure 3c and 3d shows the ability of these cells to invade through the reconstituted Matrigel ECM. HCOL1A1-transfected cells displayed a significant reduction in invasion of more than 55%.

In the *in vitro *invasion model using reconstituted basement membrane wafers, HCOL1A1-I cells hardly invaded the Matrigel wafer on day 7 (Fig. 3e). In contrast, a number of Mock cells invaded the Matrigel wafer. Thus, it was clearly demonstrated that the HCOL1A1 peptide suppressed the cell motility and invasion of T98G cells.

### Inhibition of tumor formation *in vivo *by HCOL1A1

**Figure 4:**
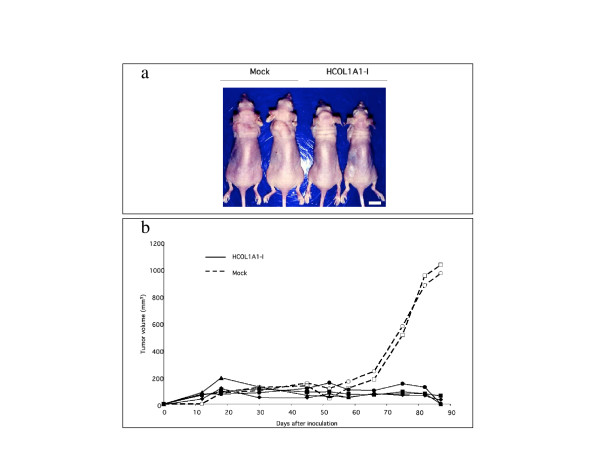
Inhibition of tumor formation of T98G cells *in vivo *by introduction of the HCOL1A1 gene. T98G cells were implanted s.c in the forelegs of nude mice, and the tumor volume was measured at each time point. (a) Tumor formation in nude mice at 87 days after inoculation with Mock cells (Mock) or HCOL1A1-transfected cells (HCOL1A1-I). Scale bar, 1 cm. (b) The *in vivo *tumor growth of HCOL1A1-transfected cells (HCOL1A1-I, n = 4) was compared with that of Mock cells (Mock, n = 2).

To assess the effects of forced HCOL1A1 expression on T98G glioma cell growth *in vivo*, HCOL1A1-I cells and Mock cells were injected into nude mice s.c., and the tumor volumes were determined 87 days later (Fig. 4). Figure 4b shows that HCOL1A1-I cells had grown more poorly and formed smaller nodules than the Mock cells. At 87 days, the animals were sacrificed, and complete tumor regression was observed in the mice transplanted with HCOL1A1-I cells. In contrast, the control cell-transplanted animals had developed large tumors of around 1,000 mm^3^. These results suggest that the introduction of HCOL1A1 was able to function as a growth suppressor in human glioma cells *in vivo*.

### Induction of apoptotic cells *in vitro *by HCOL1A1

**Figure 5:**
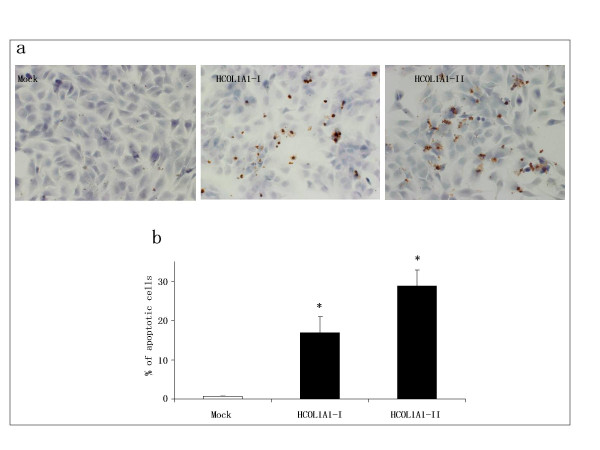
Induction of apoptotic T98G cells *in vitro *by overexpression of HCOL1A1. Mock cells (Mock) and HCOL1A1-transfected cells (HCOL1A1-I and HCOL1A1-II) were assayed for apoptosis using the *in situ *TUNEL method. (a) The photomicrographs of Apoptotic cells. Magnification, × 200. (b) The percentage of apoptotic cells represents the mean ± SD (n = 5, duplicate experiment). *, *P *< 0.001, when tested against the Mock.

The effect of overexpressed HCOL1A1 on the apoptosis of glioma was studied by the TUNEL assay (Fig. 5). Figure 5a shows that numerous apoptotic cells were detected in HCOL1A1-transfected cells, whereas apoptotic cells were scarcely seen in the Mock cells. The number of apoptotic cells was counted in the microscopic fields, and the percentage of apoptotic cells is revealed in Figure 5b. The apoptosis rates of HCOL1A1-I and HCOL1A1-II were 17% and 29%, respectively. In contrast, Mock cells showed less than 1% of apoptotic cells. Therefore, the over-expression of HCOL1A1 induced the apoptosis of T98G glioma cells and this may partially contribute to the growth suppression of HCOL1A1 tumors in mice.

## Discussion

The present study shows that the overexpression of HCOL1A1 in T98G malignant glioma cells markedly changed the cell morphology and significantly suppressed the motility and invasion of cells. In addition, the inhibition of glioma cell growth was indicated *in vitro *and *in vivo*; in particular, tumorigenicity had regressed completely in nude mice. Moreover, HCOL1A1 induced the apoptosis of glioma cells, and this finding suggested that apoptosis participates in a mechanism for the suppression of malignancy of T98G glioma cells by forced expression of HCOL1A1. Interestingly, overexpression of HCOL1A1 in 293 cells, which are transformed human embryo kidney cell line and do not express HCOL1A1, showed no influence on the cell morphology, growth and apoptosis induction (data not shown). This finding suggests that ectopic forced expression of HCOL1A1 protein in cells dose not cause of unfolded protein response which induces apoptosis, however, the effects of accumulation of unfolded HCOL1A1 protein on various cell types should be further considered.

It has been pointed out that loss of ECM control is a characteristic feature in malignant tumor progression toward invasion [19]. In glioma cells, the expression of ECM components, such as decorin, tenascin, vitronectin, laminin, fibronectin, type I collagen, type IV collagen, neuronal cell adhesion molecule (NCAM), N-cadherin, and beta-catenin, dramatically changes during tumor progression, and these ECM proteins have been reported to play a significant role in the migration and invasion of gliomas [18,20-29]. As an example of glioma therapy using ECM protein, decorin, which is poorly expressed by glioma cell lines, was ectopically expressed in glioma cells and successfully used to abrogate the growth of experimental gliomas [20,30,31]. Similarly, the overexpression of type I collagen should be useful for suppression of glioma progress.

It has become clear that ECM proteins have unknown physiological cell functions. Recently, the biological functions of various collagen fragments have been described; fragments of type IV, type XV, and type XVIII collagen have been noted for their potential [2,3]. Type IV, type XV, and type XVIII collagen are major components of various basement membranes. Although their functional role in basement membrane architecture is well known, the NC1 fragments of these collagens are involved in the regulatory functions of various cellular events, such as angiogenesis, tumorigenesis, migration, proliferation, apoptosis, and morphogenesis, which are distinct from those of original intact molecules [2-11,32-36]. Endostatin, which is an NC1 fragment of type XVIII collagen, has been extensively studied in the angiogenesis field [4,37,38]. It has been reported that endostatin inhibits tumor cell invasion, as well as HCOL1A1 peptides, which were presented here. Endostatin inhibits tumor cell invasion by blocking the activation of latent matrix metalloprotease-2, -9, and -13 [39-41]. However, in this study, there was no difference in the level of activated MMP-2 and MMP-9 between HCOL1A1-transfected cells and Mock cells by gelatin zymography analysis (data not shown). This finding suggests that the HCOL1A1 peptide may have a unique function as a suppressor of tumor cell invasion that is distinct from that of NC1 fragments of basement membrane collagens. It was demonstrated that endostatin suppresses cell proliferation *in vitro *and inhibits the growth of primary tumors and metastases by induction of apoptosis [42-44]. Similarly, the expression of HCOL1A1 peptides caused inhibition of tumor cell growth *in vitro *and complete regression of tumors in nude mice. Moreover, HCOL1A1 peptides induced apoptosis in glioma cells. Thus, HCOL1A1 peptides, as well as endostatin and other NC1 fragments of collagens, should also have a potential for anti-tumorigenesis. Interestingly, type I collagen is a fibrillar collagen, whereas fragments of collagens, which have so far been reported to inhibit tumor progression, are derived from basement membrane collagens. The HCOL1A1 peptides, which are derived from fibrillar collagen, may be novel inhibitors of tumor growth and invasion. In this report, the mechanism for the suppression of malignancy of T98G glioma is not evident, but it should become clear with further study.

Recently, significant technical advances in the treatment of gliomas have emerged, and gene therapy, in particular, is noted as a potent therapeutic strategy. The major approaches of gene therapy to glioma are based on apoptosis-related gene therapy [45], antiangiogenesis therapy [46,47], and immunotherapy [48,49]. Several ECM components and their fragments, such as decorin [20,30,31] and endostatin [46,50-54] are being tried as potential targets for glioma gene therapy. The HCOL1A1 gene may also be a good candidate as gene medicine for a novel therapy against glioma.

## Conclusion

In summary, the tumor growth and invasion of malignant human gliomas were markedly suppressed by the introduction of HCOL1A1. The suppression of a malignant phenotype of glioma cells by HCOL1A1 provides the basis of a novel therapeutic approach.

## Materials and methods

### Cell culture

The human glioma T98G cells were derived from glioblastoma and obtained from the American Type Culture Collection. Cells were maintained and passaged in a minimum essential medium (MEM) supplemented with 10% fetal bovine serum (FBS), 1% nonessential amino acids, and 1 mM sodium pyruvate at 37°C.

### HCOL1A1 expression plasmid

An α1 chain of the human type I procollagen expression vector, pCXN2/HCOL1A1, was constructed as follows. The cDNA of HCOL1A1 was cloned from a human heart cDNA library, and a partial cDNA fragment was synthesized by RT-PCR using mRNA of a normal human skin fibroblast. cDNA encoding the full-length HCOL1A1 gene was assembled from these fragments. The full-length HOCL1A1 cDNA was cloned into the downstream of the CAG promoter of a pCXN2 expression vector containing the neomycin resistance gene.

### Cell transfection

Cells were transfected with pCXN2/HCOL1A1 or pCXN2 without an insert, as a Mock, by using LipofectAMINE 2000 (Invitrogen Corporation, CA) according to the manufacturer's protocol. Cells were selected in a medium containing 0.8 mg/ml of G418. G418-resistant colonies were cloned and expanded.

### Immunocytochemical staining

T98G cells (1 × 10^5^) were grown for 2 to 4 days on 6-cm culture dishes and fixed with cold methanol at -20°C for 5 min. The cells were permeated with 0.02% Triton X-100 in PBS for 15 min and pretreated with 3% H_2_O_2 _in methanol for 15 min to quench endogenous peroxidase activity. The cells were blocked with Block Ace (Dainippon Pharmaceutical Co., Ltd., Osaka, Japan) overnight at 4°C and incubated with polyclonal anti-α1(I) collagen antibody (L-19) (1:60) (Santa Cruz Biotechnology, Inc., CA) at 37°C for 1 h. Bound primary antibodies were labeled with biotinylated IgG antibodies (1:500) (Santa Cruz Biotechnology, Inc., CA) at 37°C for 15 min and incubated with streptavidin-peroxidase at 37°C for 20 min. HCOL1A1 peptides were visualized with diaminobenzidine, and nuclei were counterstained with hematoxylin.

### *In vitro *growth assay

Individual clones were seeded in 96-well plates at a density of 5 × 10^2 ^cells per well in 100 μl of a culture medium. At each time point, the cells were assayed for proliferation with TetraColor One, a cell-proliferation assay reagent (Seikagaku Co., Tokyo, Japan), according to the recommended method; they were then measured for absorbency at the well at 450 nm with a reference wavelength at 650 nm.

### *In vitro *invasion and motility assay

*In vitro *invasion assays were performed using a Matrigel invasion chamber (8-μm pore size, Becton Dickinson, Bedford, MA). A suspension of 2.5 × 10^4 ^cells in 0.5 ml of a serum free medium, Cosmedium 001, was added to the Matrigel chamber. The chambers were incubated at 37°C for 24 h in a 95% air/5% CO_2 _incubator. The cells on the lower surface of the membrane were stained with Diff-Quik stain (Kokusaisiyaku, Kobe, Japan). The invadin cells were photographed under a microscope at × 100 magnification and counted in five fields of four membranes.

A cell motility assay was performed in a similar manner except that the 8-μm pore size PET membrane was not coated with Matrigel.

### Tumor invasion into a matrigel wafer

The reconstituted basement membrane wafers were made by adding 1 ml of Growth Factor Reduced Matrigel (Becton Dickinson, MA) to a well of a 24-well plate and gelled at 37°C for 30 min. 1 × 10^5 ^cells were plated onto the surface of each wafer. On days 3 and 7 after plating, Matrigel wafers and adherent cells were fixed with 4% paraformaldehyde in PBS for 1 h. The wafers were then dehydrated through a graded ethanol series and embedded in paraffin. Sections were cut and stained with hematoxylin and eosin.

### *In vivo *tumor formation

T98G cells (4.3 × 10^6^) were implanted subcutaneously in 100 μl of a 1:1 mixture of a culture medium and Growth Factor Reduced Matrigel in the forelegs of the nude mice according to the method described by Rubenstein *et al*. [55] and Teicher *et al*. [56]. The tumor volume was measured with a caliper and calculated using the formula width^2 ^× length × 0.5. Animal experiments in the present study were performed in compliance with the guidelines of the Institute for Laboratory Animal Research, National Cancer Center Research Institute.

### Apoptosis assay

In the normal growth medium, 1.8 × 10^5 ^cells were seeded onto 6-cm culture dishes. After 24 h, the cells were rinsed and cultured in a serum-free medium, Cosmedium 001, which contained no protein except insulin and transferrin and was supplemented with sodium ascorbate (50 μg/ml) to avoid the effects of several ECM proteins carried by the serum. Five days later, the cells were assayed for apoptosis by the TUNEL method with the *In Situ *Cell Death Detection Kit, POD (Roche, Switzerland) according to the manufacturer's instructions. Apoptotic cells were identified by diaminobenzidine staining, and nuclei were counterstained with hematoxylin. The number of apoptotic cells was counted in five microscopic fields.

### Statistical analysis

The results are given as means ± SD. Student's t-test was performed for statistical evaluation, with *P *< 0.05 considered significant.

## Abbreviations

ECM, extracellular matrix; HCOL1A1, human collagen type I α1.

## Authors' contributions

KH carried out most of experiments. KH, TM and TO participated in its design and helped to the draft the manuscript. All authors read and approved the final manuscript.
